# A64 HUMAN INTESINAL ORGANOIDS AS A MODEL FOR GASTROINTESTINAL FUNCTION AND TOXICITY SCREENING

**DOI:** 10.1093/jcag/gwad061.064

**Published:** 2024-02-14

**Authors:** M Stahl, D Leung, W Anderson, V Conlin, A Eaves, S A Louis, R K Conder

**Affiliations:** Research and Development, STEMCELL Technologies Inc, Vancouver, BC, Canada; Research and Development, STEMCELL Technologies Inc, Vancouver, BC, Canada; Research and Development, STEMCELL Technologies Inc, Vancouver, BC, Canada; Research and Development, STEMCELL Technologies Inc, Vancouver, BC, Canada; Research and Development, STEMCELL Technologies Inc, Vancouver, BC, Canada; Research and Development, STEMCELL Technologies Inc, Vancouver, BC, Canada; Research and Development, STEMCELL Technologies Inc, Vancouver, BC, Canada

## Abstract

**Aims:**

Drug-induced gastrointestinal (GI) toxicity is a common adverse event in clinical trials, with symptoms including diarrhea, ulceration, and inflammation resulting from impaired barrier function. Furthermore, the intestine is a key mediator of both drug absorption and metabolism. The human colon carcinoma cell line (Caco-2) is the most common model used in drug discovery, however, intestinal organoids provide a model that better recapitulates the self-renewal, cellular composition, and function of the intestinal epithelium.This study utilizes human intestinal organoids as a high-throughput model for evaluating intestinal function and toxicity in preclinical drug discovery.

**Methods:**

To assess toxicity, intestinal organoids were treated with two GI-toxic drugs, gefitinib and colchicine, and post-treatment cell viability was compared to Caco-2 control cultures. To assess barrier function, organoid-derived monolayers cultured in Transwell® plates were treated with colchicine and sapitinib, and post-treatment permeability was evaluated using the fluorescent low-permeability markers, 4 kDa FITC-dextran and Lucifer yellow.

**Results:**

Human intestinal organoid cultures exhibited 14-fold greater sensitivity to gefitinib (average organoid IC50 = 0.82 μM, n = 3; Caco-2 IC50 =11.4 μM, n = 2) and 2,238 times greater sensitivity to colchicine (average organoid IC50 = 0.028 μM, n = 3; Caco-2 IC50 = 62.89 μM, n = 6) compared to Caco-2 controls. This 96-well assay was reproducible, producing comparable IC50 concentrations in repeated experiments with cells from the same and different donors (n = 3 donors). Organoid-derived monolayers exhibited ampersand:003E 10-fold (n = 3) increase in permeability to both sapitinib and colchicine compared to Caco-2 cultures, which displayed minimal responses to either drug treatment. Finally, organoid-derived monolayer cultures in transwell® plates can be utilized to quantify drug transport and metabolism by the intestinal epithelium. Using ileal organoid-derived monolayers and Caco-2 cultures, we quantified the active transport of methotrexate by the efflux transporter BCRP, calculating an efflux ratio of 11.46 from the basal to apical chambers. We also quantified CYP3A4 activity of ileal monolayers relative to Caco-2 cultures. Ileal monolayers converted midazolam to hydroxymidazolam ampersand:003E 50-fold (n = 2) more than Caco-2 cultures, which displayed minimal CYP3A4 activity below the limit of detection. Together, these data demonstrate that human intestinal organoids are a valuable tool for high-throughput GI toxicity screening and for evaluating intestinal function in preclinical drug discovery.

**Conclusions:**

Together, these data demonstrate that human intestinal organoids are a valuable tool for high-throughput GI toxicity screening and for evaluating intestinal function in preclinical drug discovery.

**Funding Agencies:**

None

